# An Evidence-Based Review of Impacts, Strategies and Tools to Mitigate Urban Heat Islands

**DOI:** 10.3390/ijerph14121600

**Published:** 2017-12-19

**Authors:** Walter Leal Filho, Leyre Echevarria Icaza, Victoria Omeche Emanche, Abul Quasem Al-Amin

**Affiliations:** 1 HAW Hamburg, Faculty of Life Sciences, Research and Transfer Centre “Sustainable Development and Climate Change Management”, Ulmenliet 20, D-21033 Hamburg, Germany; walter.leal@haw-hamburg.de (W.L.F.); VictoriaOmeche.EMANCHE@haw-hamburg.de (V.O.E.); 2 School of Science and the Environment, Manchester Metropolitan University, Chester Street, Manchester M1 5GD, UK; 3 Faculty of Architecture and the Built Environment, Delft University of Technology, Julianalaan 134, 2628 BL Delft, The Netherlands; 4 Institute of Energy Policy and Research (IEPRe), Universiti Tenaga Nasional (UNITEN), 43000 Kajang, Malaysia; abulquasem@cust.edu.bd

**Keywords:** climate change, urban heat islands, cities-urban, health models

## Abstract

The impacts of climate changes on cities, which are home to over half of the world’s population, are already being felt. In many cases, the intensive speed with which urban centres have been growing means that little attention has been paid to the role played by climatic factors in maintaining quality of life. Among the negative consequences of rapid city growth is the expansion of the problems posed by urban heat islands (UHIs), defined as areas in a city that are much warmer than other sites, especially in comparison with rural areas. This paper analyses the consistency of the UHI-related literature in three stages: first it outlines its characteristics and impacts in a wide variety of cities around the world, which poses pressures to public health in many different countries. Then it introduces strategies which may be employed in order to reduce its effects, and finally it analyses available tools to systematize the initial high level assessment of the phenomenon for multidisciplinary teams involved in the urban planning process. The analysis of literature on the characteristics, impacts, strategies and digital tools to assess on the UHI, reveals the wide variety of parameters, methods, tools and strategies analysed and suggested in the different studies, which does not always allow to compare or standardize the diagnosis or solutions.

## 1. Introduction

The attractiveness of cities and the diversity of economic and social opportunities available there, are the main drivers of the continuously increasing rate of development of urbanized areas during the last centuries. According to United Nations reports, around 54% of the world’s population has been residing in urban areas (2014 figures), in comparison to 3% in 1950. Current projections show that urbanisation may increase to 66% by 2050 [1] .By occupying only 3% of the Earth’s land [2], cities account for 60–80% of total energy consumption and 75% of carbon emissions [2]. Urbanisation means that these figures are also likely to increase.

In addition to the significant direct impact of cities on global climate change due to the substantial CO_2_ emissions they release, there are also other indirect impacts due to unsustainable consumption, pollution and waste generation. Therefore, experts point out that a growing urban population is also likely to increase the direct influence of cities on the regional and global climate [1,3]. There are also some health implications here to be considered: apart from the many links between climate change and health [4], extreme temperatures in many cities are known to be associated with many detrimental health effects [5] and often with higher mortality [6].

The urban environment is also known to have an influence on its climate as well. One of such effects is an urban heat island (UHI) [7], whose trends in Europe have been analysed by Santamouris [8]. Phenomenologically speaking, UHI can be formally defined as a metropolitan area, which is significantly warmer than surrounding rural areas [9]. The UHI is the one of the most obvious atmospheric modifications attributable to urbanisation. It occurs in settlements of all sizes in all climatic regions [10]. Heat urban island was first observed in London by Luke Howard in 1833 [11]. Today, it is the most studied climate effect of cities [10].

Heat islands can occur year-round, during the day or night [12]. However, many references indicate that during the summer in temperate cities, generally the UHI reaches its peak during the night [13,14,15,16].

The phenomenon is associated with a variety of factors, among which mention can be made to changes in runoff, the concrete jungle effects on heat retention, changes in surface albedo, changes in pollution and aerosols, and so on [17,18,19], combined with atmospheric conditions. These effects are to a large extent caused by changes in the land surface. The replacement of trees and vegetation by surfaces with less permeable materials, minimizes the natural cooling effects of shading and evaporation of water from soil and leaves-evapotranspiration-[9]. Waste heat from traffic, industries and air conditioners increase temperature and thus further exacerbate the heat island effect. Moreover, densely constructed buildings, from materials with large thermal admittance, and narrow streets, reduce air flow and natural cooling effects by holding and blocking heat from rising into the cold sky [9]. Cities also have a larger surface area compared to rural areas and therefore more heat can be stored [7]. The land use and the design of the peri-urban landscapes surrounding the cities, together with the design of the city boundaries (e.g., high buildings blocking wind paths versus green wind corridors) also influences the intensity of the phenomenon [20].

There is a perceived need to address urban sustainability matters at a regional scale [21], in order to ensure a social, economical and environmental balance between the cities and their rural surroundings [22]. However, when it comes to the assessment of climate change impacts and mitigation options, comparatively little attention is being paid to sub-regional and regional scale processes. Or to vulnerability, which is often overlooked [23]. This, in turn, leads to a reduced willingness to engage on mitigation processes, which make extreme events—when they occur—quite costly [24].

Furthermore, political and social awareness often concentrate either on macro-scale climate modelling, with the main focus on greenhouse gas mitigation, or adaptation to climate change, or on disaster management where little attention is given to the exposure and responses of cities to climate change and extreme weather events [25]. As a consequence, regional climatic phenomena such as the urban heat island are not being appropriately assessed [26].

Some recent studies [27,28], have addressed the synergetic reactions of heat waves, and a study performed by Founda and Santamouris [29] explored the synergies between UHI and heat waves in Athens (Greece), during an extremely hot summer (2012). UHIs, when coupled with increased population density, tend to exacerbate the many problems already seen in urban settings.

UHIs have multiplied effects on climate and urban dwellers, mainly on local level. It increases summertime peak energy demand that elevates emissions of air pollutants and greenhouse gases, which in turn contribute to an UHI of greater magnitude [12,30]. Increased emissions lead to lower air quality compromising human health [30]. UHIs also impair water quality. Warm water ends up flowing into local streams: rivers, ponds, and lakes that stresses the native species that have adapted to life in a cooler aquatic environment [12,30]. Keramitsoglou et al. [31] outlined the roots of urban thermal risk reduction whereas [32] offered a detailed urban heat island projections for some cities.

However, the key issue is the effect of urban heat island on urban climate and surroundings. Consistent with the need to investigate this important topic, the aim of this paper is to analyse the different parameters, scales, intensities, health impacts, mitigation proposals and diagnosis tools, used to assess the UHI phenomenon across the globe, in order to highlight the huge disparity of methods, which can complicate the transferability of the results. The study had a limitation in the sense that the availability of reliable data in many cities-especially but not only in the developing world—means that records of UHI are either unreliable or non-existing. Data provided in the paper has thus been carefully scrutinized and is provided on occasions there are assurances they are robust.

## 2. Materials and Methods

In order to understand how consistent the different studies are, how comparable the results, and how formalised the procedures of analysis and tools, this study analyses and classifies UHI literature in three stages:

- Characteristics of the phenomenon in different cities across the world, identifying parameters analysed and mitigation proposals suggested based on the analysis.
- Analysis of different UHI mitigation strategies proposed, and description of the principle and effects.
- Analysis of digital UHI online tools, identifying geographical cover, scale of assessment, type of assessment and limitations.

## 3. Results

### 3.1. Trends on Urban Heat Islands in Cities Round the World

#### 3.1.1. The UHI in the World

Urbanisation impacts the climate on both regional and local levels. It results in differences between a city and rural area in cloud cover, precipitation, solar irradiation, air temperature and wind speed. The geometry, spacing and orientation of buildings and outdoor spaces strongly influence the microclimate in the city [7]. Perturbation of surface energy balance caused mainly by reduction of evaporative cooling, release of anthropogenic heat and increase of solar radiation input due to decreased albedo. All these contribute to UHI [33], which in turn exacerbate the impact of heat waves, periods of abnormally hot, and often humid, weather [30]. However, UHIs might also have positive effects in cities in cold climates, namely, a smaller number of snowfall and frost events, longer growing season as well as reduced energy demand for domestic heating [10].

UHI affects cities worldwide. So far, a worldwide city classification system which would help implement semi-customised UHI mitigation measures, is yet to be developed. However, there are well documented worldwide studies on parameters influencing the UHI. Below an overview of the worldwide assessment of UHI-related parameters.

**Figure 1:**
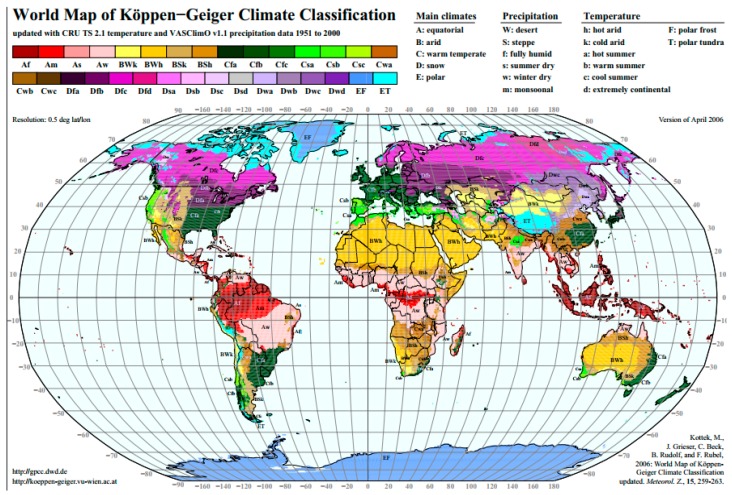
World map of Köppen-Geiger climate classification [34].

**Figure 2:**
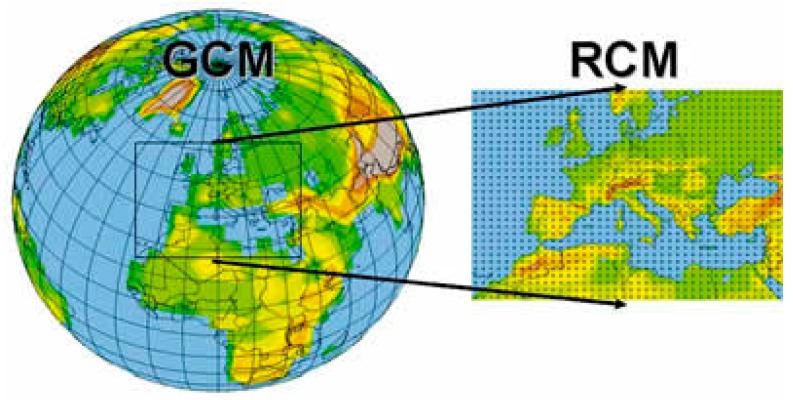
Downscaling from Global Climate Models (GCMs) to Regional Climate Models (RCMs) [36].

**Figure 3:**
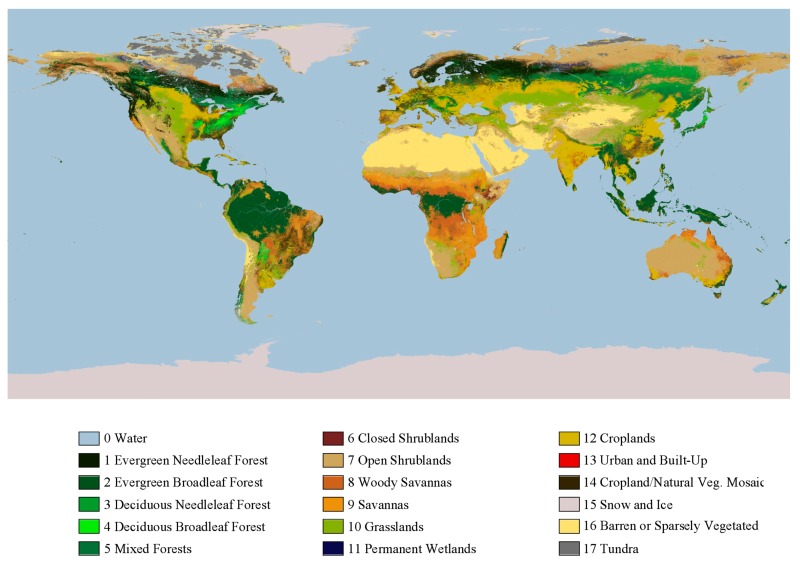
Color-coded land cover classification differentiating 17 types of land cover, ranging from evergreen needleleaf forest to tundra [41].

**Figure 4:**
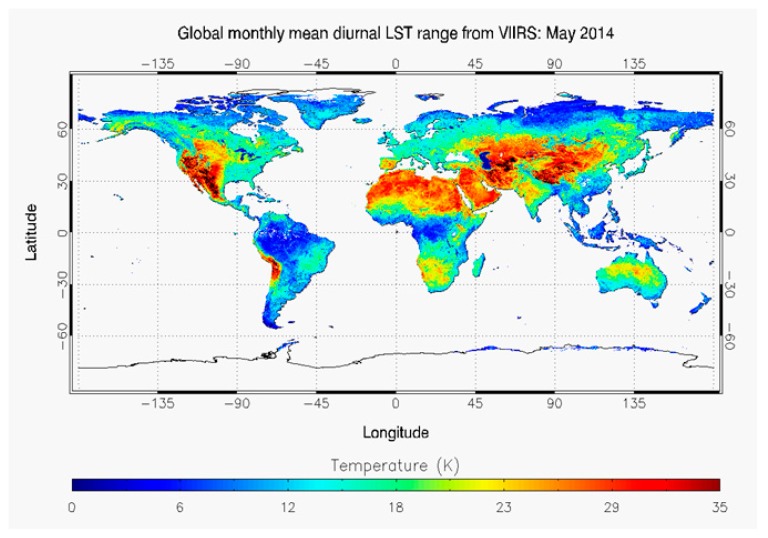
Global Monthly Mean from May 2014 of diurnal range of LST from S-NPP VIIRS [45].

**Figure 5:**
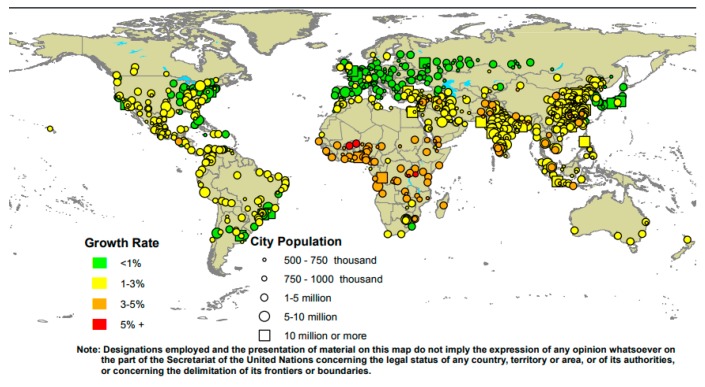
Growth rates of urban agglomerations by size class [1].

**Figure 6:**
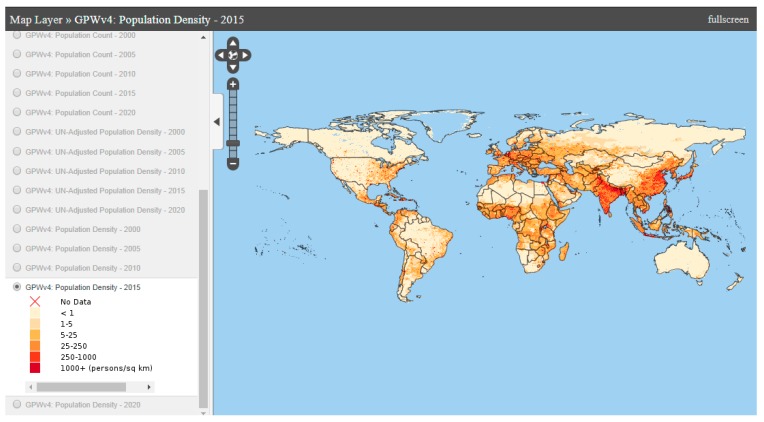
Population density 2015 [47].

- Parameters related to the geographical location:
  ○ Climate classification: Köppen-Geiger climate classification (presented by Köppen in 1900, and updated by Geiger in 1954 and 1961) is still the most frequently used climate classification [34] (Figure 1). It provides a good overview of the earth’s climates which inevitably influence the UHI [33], however, other parameters should also be taken into consideration as well [35].
  ○ Global and regional climate models: The global and regional climate models are atmospheric simulations meant to predict the impact on climate of greenhouse gas emissions. If climate influences UHI, the study of future climate conditions is also relevant for the analysis of the phenomenon (Figure 2) [36].
  ○ Land cover classification: Several studies show the relationship between land cover and UHI [37,38,39,40]. NASA’s Goddard Space Flight Center produced a worldwide land cover map using satellite imagery and enhanced models (Figure 3) which provides a good basis for UHI assessment worldwide.

- Parameters related to the urban environment:
  ○ Local climate zones (LCZ): The World Urban Database and Portal Tool (WUDAPT) is a project that aims at retrieving, storing and disseminating data on physical characteristics of cities worldwide [42]. It acquires data related to form (surface cover, the construction materials and geometry) and function (metabolism, i.e., exchange of energy, water and materials) of cities [43]. It uses the Local Climate Zones [44] which classify neighbourhoods based on their influence on local air temperature, to produce a method for a more refined worldwide systematic classification assessment.
  ○ Land surface temperature: Satellite imagery also allows to map land surface temperature worldwide [45] (Figure 4). Land surface temperature is often used to characterize UHI [46,47].
  ○ City sizes. Several studies relate the size of the cities to the magnitude of the UHI [48,49,50]. Others highlight the role of more relevant parameters [18,51]. The United Nations has mapped the size of cities and their corresponding growth rates [1] (Figure 5).
  ○ Urbanisation predictions: Future prospects for urbanization are also important data to assess the evolution of the phenomenon at medium term (Figure 5).
  ○ Population density: Several studies establish a relationship between population density and UHI [52,53]. The United Nations Socioeconomic Data and Applications Center (SEDAC) produced the The Gridded Population of the World (GPW) series which consist of a set of maps which model the distribution of human population (counts and densities) on a continuous global surface of censuses occurred between 2005 and 2014 (Figure 6).

The data related to the climate and natural environment is mostly retrieved from satellite imagery, and is thus mostly already available. In turn, the data on the urban environment requires the combination of other tools. More specifically, the LCZ mapping requires more sophisticated processing [43] and as a matter of fact the WUDAPT is still being developed. The overlap of these parameters should provide a good basis for the development of a worldwide city classification system for the UHI assessment.

#### 3.1.2. Examples of Causes and Impacts of the UHI in Cities Worldwide

This section documents examples of the causes and impacts of the urban heat island effect in different cities around the globe (Table 1).

##### Example 1: USA

The mean decadal increase of the UHI of large US cities between 1951 and 2000 has been 0.05 °C [54]. According to the EPA, many U.S. cities have air temperatures up to 5.6 °C warmer than the surrounding natural land cover [55]. Analysis of the regional temperature trends calculated from seven long-term observation stations for the summer and winter seasons between 1950 and 2014 identified an UHI effect in Reno, Nevada that is maximized during summer (June–August) [56]. Debbage and Shepherd 2015 estimated the urban heat island intensities of the 50 most populous cities in the United States. Their findings indicate that the spatial contiguity of urban development, regardless of its density or degree of sprawl, is a critical factor that influenced the magnitude of the urban heat island effect. An increase of 10 per cent of urban spatial contiguity might enhance the minimum temperature annual average UHI intensity by between 0.3 and 0.4 °C [57].

##### Example 2: UK

Kershaw et al., 2010 estimated the heat island effect in the UK cities. The obtained results showed that the UHIs are largest in the summer, but not for every city. For example, in small cities such as Leicester or York, the UHIs are very small and do not vary with season. The annual average UHIs for different UK cities range between 0.1 and 1.9 °C, whereas in a summer period between 0.1 and 2.0 °C, for example, in London it varies between 1.6 and 1.9 °C for summer [16]. In London, since the 60’s the nocturnal UHI in spring and summer has increased approximately 0.12 °C/decade. The projections foresee a further increase of 0.26 °C by 2080 [58].

##### Example 3: Belgium

Lauwaet et al., 2016 examined the urban heat island of the country’s capital, Brussels, for 2000–2009 and projected 2060–2069 climate conditions. The obtained results indicate that the presence of the urban heat island has an impact on extreme temperatures, especially during the night. Such temperatures are also expected to occur more frequently in the future. At the same time the authors project only very small change in the magnitude of Brussels’ UHI in the near future. Overall, the mean night-time UHI of Brussels accounted to 3.15 °C for the 2000–2009 [59].

##### Example 4: The Netherlands

Icaza et al. [19] performed a comparative analysis of heat related parameters (storage heat flux, vegetation index, land surface temperature, albedo, sky view factor and coolspots) retrieved through satellite imagery analysis of six Dutch cities: The Hague, Delft, Leiden, Gouda, Utrecht and Den Bosch. The analysis revealed that the hotspots of the six cities were located in the seventeenth century city centres. The increase of the albedo of all roof surfaces comprised within the hotspot boundaries was estimated to reduce the UHI effect by 1.5 °C. For the cities of The Hague, Delft and Leiden the maximum UHI (based on hobby meteorologists data) was estimated to range from 4.8 °C till 5.6 °C [51]. The UHI of the city of Amsterdam was analysed by Van der Hoeven and Wandl 2015 who produced a set heat related maps: landuse, imperviousness, social vulnerability, building vulnerability. The nocturnal air temperature images present air temperature differences of above 7 °C [60].

##### Example 5: Greece

Greece’s capital, the city of Athens, is characterised by strong heat island effect, mainly caused by its geographical position and its accelerated industrialisation and urbanisation during the last decades. The annual mean air temperature difference between the urban and the rural stations increased at a rate of 0.2 °C/decade [61]. The UHI in the city is mainly linked to limited green and open space areas, lack of water evaporation, the high heat storage capacities of building and surface materials, air pollution as a result of dense traffic and nearby industries, and intense air conditioning [62]. According to Santamouris et al., 2007, the ecological footprint of the additional CO_2_ emissions caused by the presence of the heat island effect ranges 1.5–2 times the Athens’s political area of 2.929 km^2^, whereas the maximum potential ecological footprint, provided that all buildings are air conditioned, is approximately 110,000 hectares [63].

##### Example 6: Germany

An example from Germany is provided by the city of Stuttgart, located in the Neckar basin, surrounded by steep hill slopes, and with an area of 207.4 km^2^. The city is located at around 240 m above sea level, whereas the surrounding hills reach up to 500 m. This particular topography worsens the UHI effect as well as the air quality. The mean annual temperature will be increased by 2 °C in the climate projections for 2071–2100 and the Great Stuttgart region will experience more than 30 days heat stress by 2100 [64]. In such context, preserving and enhancing existing green infrastructure surrounding the city becomes critical. The Climate Atlas of the Region of Stuttgart is one of the best-known examples of integrating climate knowledge into spatial planning. The city of Stuttgart has around 600,000 inhabitants, and its metropolitan region has around 2,600,000 inhabitants. This is primarily an industrial region and has had a long tradition of air quality concern, which is probably the triggering factor for its climatic awareness. Its 2008 Climate Atlas City of Stuttgart [65] highlights cold production areas, air catchment areas, as well as different breeze systems. The most important cold production green infrastructure area is located near to the western part of the city, and has a surface area of around 1000 ha. One important characteristic of this climate buffer is that it is actually connected to a larger territorial landscape between the cities of Leonberg, Sindelfingen, Vaihingen and Boblingen.

##### Example 7: Malaysia

Morris et al., 2015 investigated the existence and distribution of UHI in the administrative capital of Malaysia, Putrajaya, with a population of over 88.000 inhabitants. The city is built on the garden-city concept. The obtained results have shown that UHI intensity of Putrajaya varies temporally and spatially. It increases during the night to a peak value and then diminishes in the morning with a negligible value during mid-day. During the night the UHI ranges from 1.9 °C to 3.1 °C. The overall effect of urbanized local climate zones heating of Putrajaya is normalized by the total amount of area reserved for vegetation [66].

##### Example 8: India

Borbora and Das 2014 assessed the urban heat Island intensity (UHII) during the summertime in Guwahati, a small but rapidly growing city of India, where humidity conditions are high. The findings show the existence of UHII above 2 °C. The highest magnitude of daytime urban UHII accounts to 2.12 °C while highest night-time UHII to 2.29 °C. They also found that that the formation of daytime UHII of =1.5 °C is fairly common. Therefore, the authors conclude that with incremental decrease in green cover associated with urbanization, will enhance the UHI phenomenon that will result in substantially higher level of discomfort for dwellers [67].

##### Example 9: Japan

According to Fujibe 2011, in some cities in Japan, the increase in annual extreme minimum temperature exceeds 10 °C/century. The studies also revealed widespread urban warming (the extended heat island) around Tokyo and other megacities such as Osaka and Nagoya in the afternoon in summer. The obtained results are explained by the enhanced surface heating over a large urban area, and a reduction of sea breeze penetration caused by increased surface convergence. An analysis of meteorological data indicated the existence of anomalous temperature changes. Locations with population density of 100–300 per km^2^ has the anomalous trend of 0.04 °C/decade [68].

Some trends are also seen in Africa [69] where efforts to handle the problem are seen. Indeed, numerous mitigation strategies are being employed as a response from city authorities across the world to the adverse effects caused by the urban heat island phenomenon. Some of them are introduced in the next section of this paper.

### 3.2. Strategies to Reduce Heat Islands

By diminishing the accumulation of heat and applying cooling techniques, cities can reduce the temperature difference between urban and rural areas [70]. There have been several attempts to produce catalogues describing not only the causes and impacts of UHI, but also suggesting mitigation strategies:

- In Quebec the Urban Heat Island Mitigation Strategies catalogue [71] organizes the mitigation strategies around four sections:
  ○ Vegetation
  ○ Sustainable urban infrastructure
  ○ Sustainable stormwater management
  ○ Reduction of anthropogenic heat

It then also classifies by scale (building and city scale) the mitigation measures. The building mitigation measures are classified in three sections: protection from solar radiation, minimization of heat infiltration, reduction of anthropogenic heat and maintaining comfortable thermal environment, whereas the urban planning and development measures are grouped in three areas: greening, urban infrastructure and reduction of anthropogenic heat.

This catalogue comprises short term mitigation measures such as ensuring the access to the so called “cooling centres” which are any air conditioned public buildings that can accommodate public (shopping centres, schools, cultural centres), the creation of air-conditioned shelters for outdoor workers [72] or even the access to aquatic facilities (including pools and misters) in natural environment or public installations [73].

- The catalogue developed within the framework of the UHI project which was implemented through the Central Europe Programme co-financed by the ERDF [74] structures the actions in four packages:
  ○ Buildings
  ○ Pavements
  ○ Vegetation
  ○ Street morphology

Its classification does not organize the mitigation actions by the immediacy of its effect (short, medium or long term effect), and in turn in its introduction a clear distinction is made between adaptation measures and mitigation measures. Adaptation measures are considered measures where the direct intervention of users is necessary—clothing, air conditioning [75]—and that do not have any positive effect on the outdoor thermal comfort, or even that have a negative one—heat released by air conditioning [76,77]. In turn, the mitigation strategies are considered well prepared and consistently applied actions. This is the reason why the mitigation measures presented include less actions than other catalogues.

- The Yamamoto compilation study [78] organizes the mitigation strategies in three blocks:
  ○ Reduction of anthropogenic heat release
  ○ Improvement of artificial surface covers
  ○ Improvement of urban structure
  and introduces important characteristics for each mitigation strategy:
  ○ Scale (individuals, buildings, ward, city)
  ○ Period (short, medium or long term)
  ○ Degree of effect (on sweltering nights or on daytime temperature rise)
  ○ And administrators of the actions (individuals, business institutions, local governments…)
- There are other catalogues that attempt to keep updated the review of the UHI mitigation literature, such as the catalogue of strategies for tropical Singapore, which focuses in improving the outdoor thermal comfort in the tropical climate [79].

The conclusion of the review of the above-mentioned catalogues is that even though the structure of the catalogues varies, there is a consensus in the nature of the UHI mitigation strategies.

A summary of these is provided below.

#### 3.2.1. At Building Scale

The local climate zone classification system [44] classifies the built environment in 10 different climate zones, from LCZ 1 to LCZ 10 (compact high-rise, compact midrise, compact low-rise, open high-rise, open midrise, open low-rise, lightweight low-rise, large low-rise, sparsely built and heavy industry). This classification system, considers the existing different urban typologies as a whole (and consider sky view factor, aspect ratio, building surface fraction, impervious and pervious surface fraction, height of roughness elements and terrain roughness class. Thus the imperviousness assessment does not distinguish between pavement or roof, and albedo is also not taken into consideration. High resolution GIS maps and satellite imagery would help provide the UHI assessment at building scale.

##### Choice of Roofing Materials

The choice of roofing colour can contribute to temperature reduction by approximately 12%. Roofing materials with high albedo reduce absorbed solar heat and make a house less warmer that results in lower energy consumption [80]. Studies in Singapore demonstrated that the city-scale deployment of cool roofs can greatly reduce the near-surface air temperature and surface skin temperature during the daytime [81].

##### Use of Green Roofs

The wide conversion of the black roofs into green roofs can have positive effects on micro—and urban scale as well provide better storm-water management—improving water retention by 7% to 10%, also leading to improvements of air quality and increases in urban biodiversity [82].

##### Reduction of Anthropogenic Heat Production

Anthropogenic heat generated by exhaust heat from outdoor AC units also contributes to the formation of UHI. Appropriate nocturnal cross ventilation, window shading, appropriate building insulation [83] or the implementation of green roofs help decrease the use of energy for cooling and heating by between 20% and 25% depending on the construction materials used and whether or not green roofing is being used.

The use of geothermal energy and radiant cooling systems, are alternative solutions to conventional air conditioning systems, which contribute to the reduction of anthropogenic heat.

#### 3.2.2. At City Scale

A worldwide city classification system would help consider the parameters below by classes. It would also include parameters such as city size, population density, climate zone, … (see parameters described in Section 3.1.1), which all influence the UHI. Some of these parameters are not taken into consideration in the mitigation proposals catalogues.

##### More Urban Green Vegetation

Vegetation provides shade, thermal insulation to keep the interior cool, manage noise and air pollution [10]. According to Takebayashi and Moriyama 2007, the sensible heat flux is small on the green surface [84]. Vegetation reduces the near-surface air temperature on average by of 1–4.7 °C, particularly during night-time when the UHI intensity is high [7,81]. Assessments of the thermal load in terms of surface temperature in Tel Aviv demonstrated that the relatively low vegetation cover to free space ratio decreases the cooling effect of residential areas. Therefore, the authors recommend to ‘green’ areas within the private urban space instead of building new small-medium parks in metropolitan areas that are usually lack of free space [70]. It is also important to note that increased urban green areas might potentially be offset by increased municipal water usage, especially, in the regions with scarce water resources, e.g., in Phoenix, Arizona, where per capita residential water use is significantly higher compared to other U.S. cities [11].

##### Choice of Pavement Materials

The finishing materials of urban ground surfaces also have a major impact on the UHI effect [85]. Replacing materials with new surface cover reduces the radiative heat gain in the material by about 18–20% and improves evaporative properties of the urban surface [10]. Cool pavements have substantially lower surface temperature and reduce sensible heat flux to the atmosphere. The current research trends in the field are focused on the development of highly reflective pavements and permeable pavements that use the cooling evaporation capacity of water [86].

##### Urban Structure

Changes in building development and design options might have a positive impact on reduction of building energy use and the UHI [10]. The distribution of the buildings and urban structures in a city affect the formation of the urban heat island, since this distribution usually determines the absorption of solar radiation and the formation of air flows [85]. Designing building with considering wind properties can also lead to effective cooling of buildings in urban areas [7], reducing heat by about 25% depending on the area and properties of the building.

##### Access to Cooling Centres

These are air conditioned public buildings: shopping centres, schools, cultural centres … as discussed at the beginning of Section 3.2. These are controversial, as they provide shelter to vulnerable population, but in turn they contribute to the formation of the UHI due to the production of anthropogenic heat. Thus, they can be considered short term adaptation measures.

##### Stormwater Management Infrastructure

Retention ponds (receive runoff water and infiltrate it into the ground, can be used as green recreational areas), infiltration trenches (receive runoff water and can be easily integrated in the urban environment), dry wells (receive runoff water and are covered with gravel and sand), reservoir pavement structures (collect water at the source, through pervious finishing materials).

##### Reduction of Anthropogenic Heat

At city scale anthropogenic heat is produced either by buildings (Section 3.2.1) or by cars. Several measures can help reduce traffic anthropogenic: greener cars, improving public transit, reducing sprawl, increasing mixed-used development and by encouraging the use of electro-mobility.

#### 3.2.3. At Regional Scale

The local climate zone classification system [44] classifies the “natural” environment in seven zones, from LCZ A to LCZ G (dense trees, scattered trees, bush/shrub, low plants, bare rock or paved, bare soil or sand and water). This classification does not take into consideration functions (as described below), however the WUDAPT project intends to include functions in these classification [43]. The overlap of the local climate zones and the functions should allow to identify the elements described below which play an important part in the assessment at regional scale. This would help research, promote and implement the development of mitigation measures at regional scale, which are often not investigated enough [87].

##### More Peri-Urban Vegetation

The European Environment Agency urban adaptation document [88] suggests interventions to reinforce green infrastructure outside the city boundaries in order to manage the three main climate change phenomena threatening cities: heat waves, floods and droughts, which are projected to increase in frequency, intensity and duration [89]. Further, as far as governance is concerned, it also specifically highlights the importance of developing multi-level territorial spatial planning approaches to coordinate the responses to climate change challenges, from the city level through to national and EU levels. The regional level is the intermediate scale that links and connects cities with national territorial policies.

##### Catering for Wind Corridors

Wind corridors are designed to maximize the cool air transportation from natural cooling sources (typically green infrastructure) towards urban hotspots through advection [20]. Relevant examples of the study of the air circulation patterns applied to spatial planning are Climate Analysis Map for the Stuttgart region 2008 [65], the urban climate analysis map for the city of Arnhem [90] in the Netherlands and the ones envisioned by the Land Use Plan 2020 of the city of Freiburg in Germany [91].

##### Using the Ecological Functions of Water Bodies

Water might reduce temperature by evaporation, absorbing heat and transporting heat out of the area by moving, as in rivers. It has an average cooling effect of 1–3 °C to an extent of about 30–35 m. Such functions of water are already applied in Dutch cities [7]. Hendel et al., 2016 assessed the thermal effects of pavement-watering in Paris during the summers of 2013 and 2014. The obtained results showed that pavement-watering has UHI-mitigation effects and is an effective measure to reduce maximum daily heat stress. The maximum reduction of 0.79 °C, 1.76 °C and 1.03 °C for air, mean radiant, UTCI-equivalent temperatures and UHI-mitigation of −0.22 °C have been recorded during the day [92].

##### Land Use Considerations

The average night time land surface temperature of different land use patches varies depending on the size, shape and nature of the land use (forests, cropland, grassland, water surfaces, built areas and greenhouse areas) [20]. These should be taken into consideration when designing landscapes nearby to UHI hotspots.

#### 3.2.4. Raising Awareness among Residents

Apart from technical mitigation strategies, governments implement measures aiming directly at reduction of the climate discomfort of urban residents and their vulnerability to heat stress. The US EPA provides guidelines to the local governments to assist in the developing plans to adapt to heat. Among the main components are forecasting and monitoring, education and awareness, and heat wave response. Reliable weather forecasts allow city officials to warn citizens of heat waves in a timely manner and to prepare responses. Education and awareness efforts help to disseminate information about symptoms of excessive heat exposure and heat-related illness, recommended response and treatment, and potential risk factors [93]. Bearing in mind that the economic and environmental effects of unilateral climate actions are limited [94], these measures should ideally be implemented by groups of cities, so as to maximise their impacts.

### 3.3. Systematizing Estimation and Adaptation Measures to UHI

Several studies [95] indicate that the need for deep climatological studies on the UHI is as critical as the need for quick, multidisciplinary, high level understanding of the impact of the phenomenon to ensure it is taken into consideration since the initial stages of the spatial design. Since the options to mitigate and or adapt to the UHI phenomenon are numerous, there have been several attempts to create tools for urban planners to obtain an overview of its actual effect and potential mitigation strategies available.

#### 3.3.1. The “Decision Support System” (DSS)

The “Decision Support System” (DSS) was developed in the framework of the UHI Project, co-financed by the European Regional Development Fund. It covers 8 metropolitan areas and mega urban regions in the Central Europe Region (Bologna/Modena, Venice/Padua, Wien, Stuttgart, Lodz/Warsaw, Ljubljana; Budapest and Prague). For each of these areas, the tool provides an overview of the extent of the phenomenon, and suggests mitigation actions at two different scales: the building and the urban ones, and analyses the feasibility of the implementation of measures affecting facades, roofs, surface lots, urban structure and urban green in existing structures and new constructions [96].

**Figure 7:**
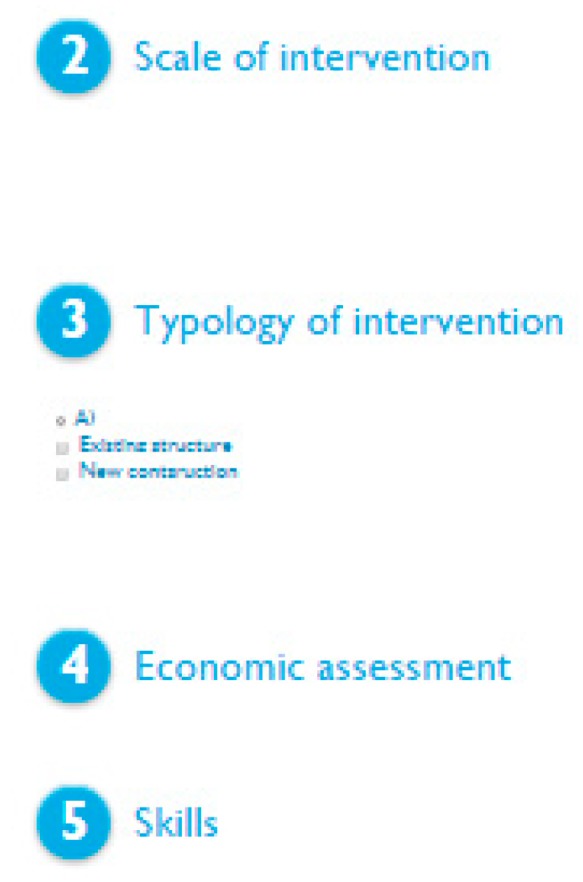
Screenshot of the interactive assessment configuration of the DSS tool.

This webpage can be considered an interactive tool for urban planners, as these first select the location in which they are interested, the scale at which they wish to intervene, an economic assessment which is actually an online calculator and a checklist of the skills on which one chooses to be assessed (Figure 7). Based on these, a customised report is issued which consists of three main elements: a climate change assessment of the selected area, a set of normative applicable to the selected area and skills, a set of potential mitigation strategies. The final part of the report is common to all reports and consists of a description of the pilot actions undertaken within the UHI project and a list with the contact details of the partners involved in the project.

**Figure 8:**
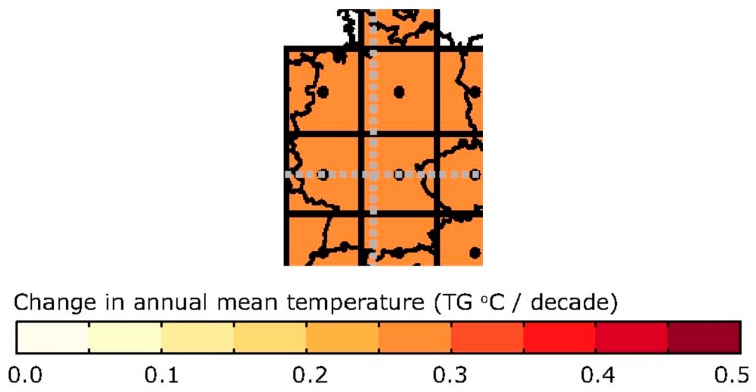
Screenshot of the customised map output showing the change in annual mean temperature per decade for the city of Stuttgart.

**Figure 9:**
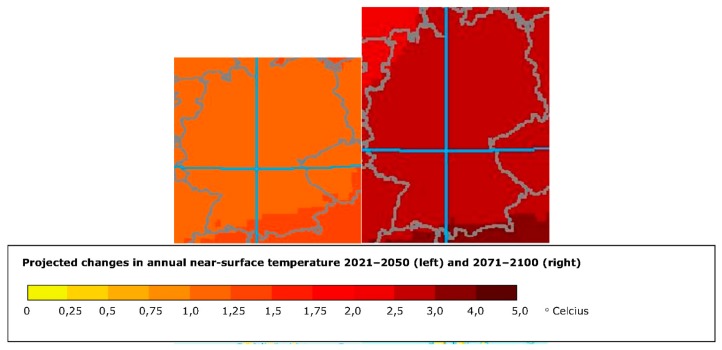
Screenshot of the customised map output showing the projected changes in the annual near-surface temperature for the periods 2021–2050 and 2071–2100 for the city of Stuttgart.

**Figure 10:**
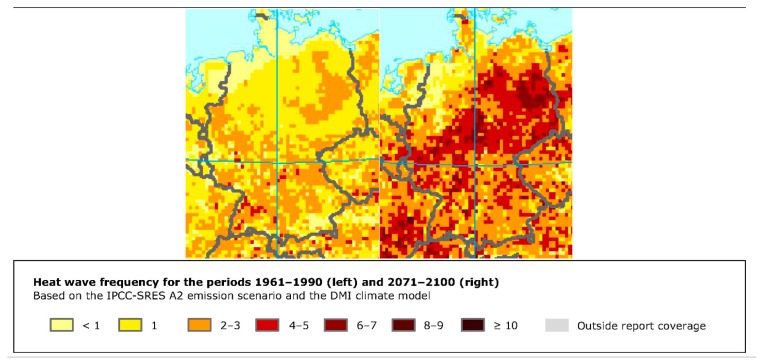
Screenshot of the customised map output showing the heat wave frequency for the periods 1961–1990 and 2071–2100 for the city of Stuttgart.

The climate change assessment of the area consists of a set of maps: one showing the change in the average annual mean temperature every decade (Figure 8), one showing the projected changes in the annual near-surface temperature for the periods of 2021–2050 (Figure 9), and 2071–2100 and finally one identifying the heat wave frequency for the period of 1961–1990 and 2071–2100 (Figure 10).

#### 3.3.2. The CE Urban Heat Island Atlas

**Figure 11:**
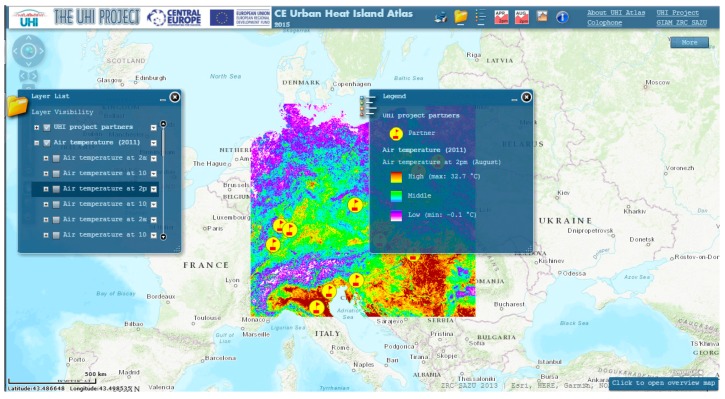
Screenshot of the CE Urban Heat Island Atlas.

The CE Urban Heat Island Atlas was also developed in the framework of the UHI Project, and is actually an interactive digital map which allows overlapping layers of parameters which play a role in the formation of the UHI phenomenon in the Central Europe region. The different layers available are: location of the project partners, air temperature, digital elevation model, land surface temperature (day and night), normalized differential vegetation index, land cover (corine), and urban atlas land use (Figure 11).

#### 3.3.3. The STAR Tools

The STAR tools was developed within the framework of Green and Blue Space Adaptation for Urban Areas and Eco Towns (GRaBS) project (The Interreg IVC EU program) which aims to improve the planning and development policy making in the context of climate change. It has been developed of the North West region of England and it includes a surface temperature tool and a surface runoff tool, which allows to estimate the impact on surface temperature and surface runoff for several land use scenarios under different temperature and precipitations scenarios [97].

**Figure 12:**
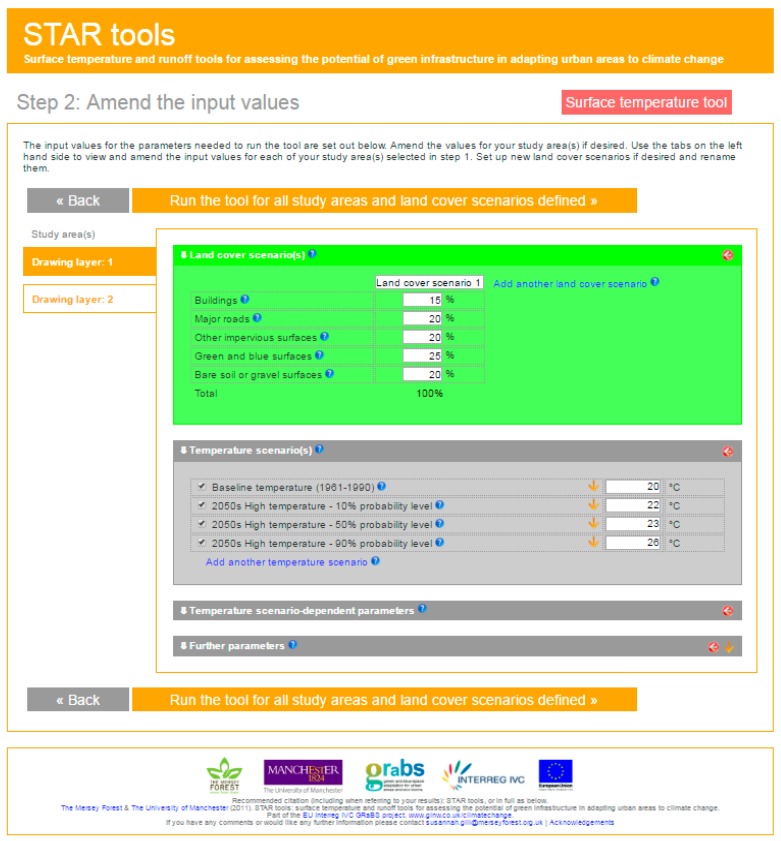
Screenshot of the input values that should be incorporated by the user, which refer to the land cover scenario for the selected area, the considered temperature scenario.

**Figure 13:**
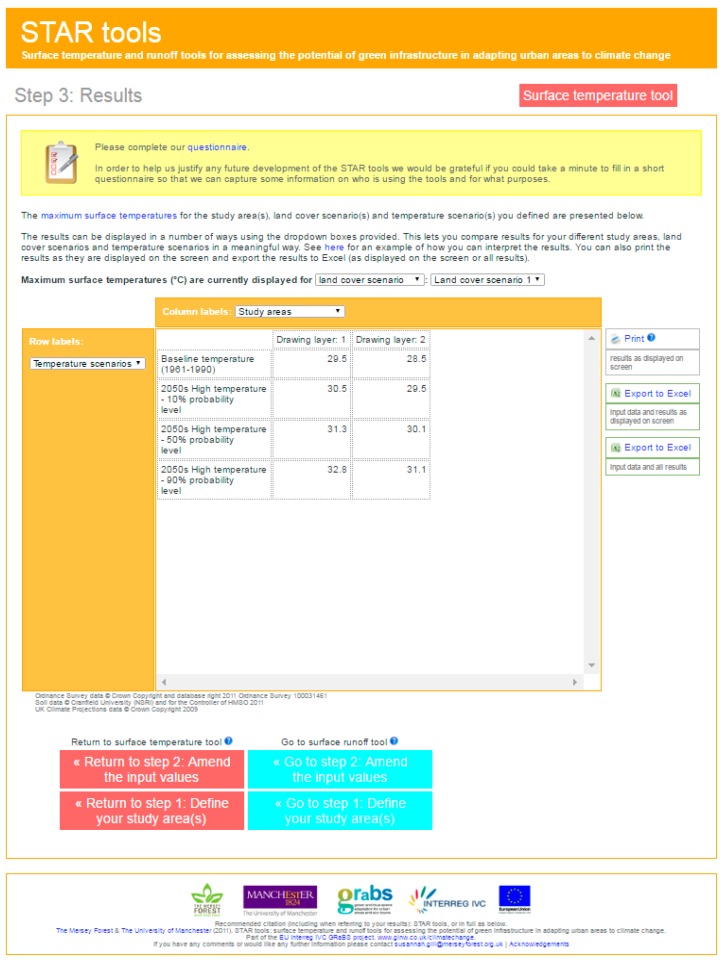
Screenshot of the input values that should be incorporated by the user, which refer to the land cover scenario for the selected area, the considered temperature scenario.

This webpage can be considered an interactive tool for urban planners, as these can select the location in which they are interested, the land cover scenario they wish to analyse (where buildings, major roads, other impervious surfaces, green and blue surfaces and bare soil or gravel areas can be differentiated) as well as the temperature scenario for the 2050’s they wish to consider (10%, 50% or 90% probability level) (Figure 12). Even though the tool can be used at different scales, it is best used at neighbourhood scale. The output of the surface temperature tool, is an exportable spread sheet (Figure 13) indicating the maximum surface temperature estimated based on the selected land cover and temperature scenario considered.

#### 3.3.4. The “London Unified Model” (Londum)

**Figure 14:**
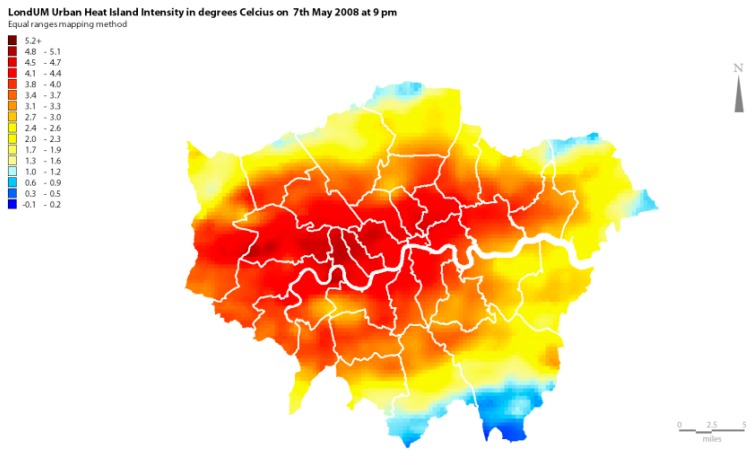
Screenshot of the output map of Londum model estimating the Urban Heat Island Intensity on May 2008 at 9.00 p.m.

The “London unified model” (Londum) developed within the Lucid program [98] is a city wide climate atmospheric model at 1 km grid, which features city wide air temperature maps at 1.5 m height. It has been developed for the city of London which estimates the impact of the volume on long and short wave radiation (reflection, shadow, conduction of heat into the building and calculation of the flux into the atmosphere) (Figure 14) [99,100].

#### 3.3.5. The ADMS Model

**Figure 15:**
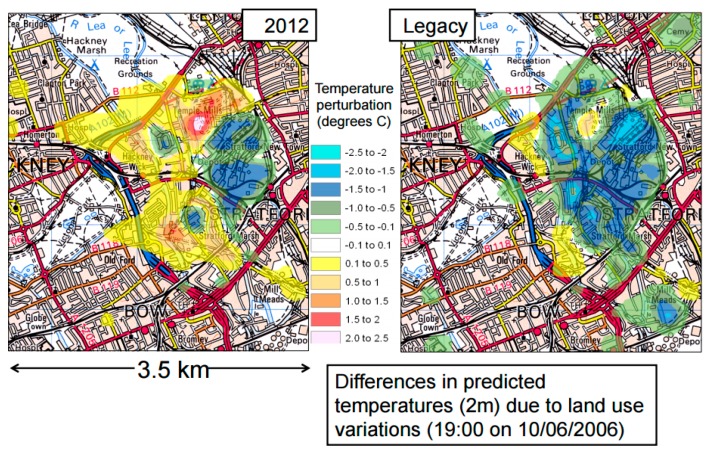
Screenshot of the output map of ADMS model estimating the temperature changes due to land use variations on the Olympic Parkland site development.

The Atmospheric Dispersion Modeling System (ADMS model) is an atmospheric dispersion model which was also developed within the Lucid program for the city of London and which features the perturbation on temperature and humidity at a neighbourhood scale model based on the building volume and surface covers (it considers albedo, evapotranspiration and the thermal admittance of surfaces) (Figure 15) [99,100].

#### 3.3.6. The LSSAT

**Figure 16:**
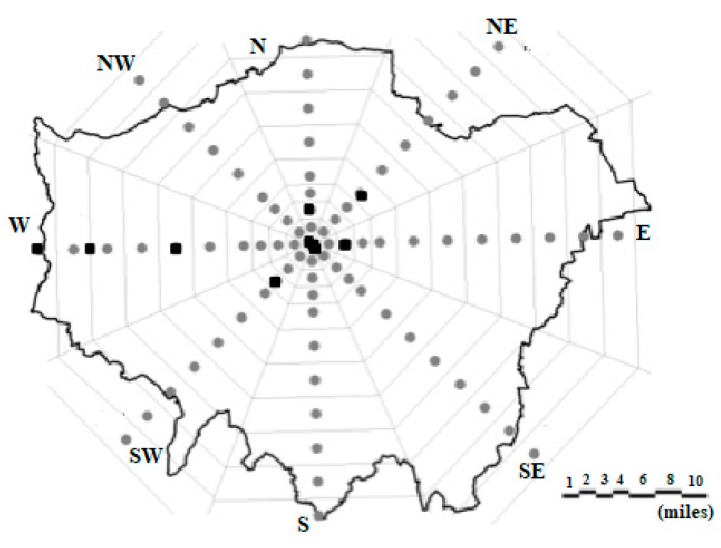
LSSAT model, fixed temperature stations along the eight transects of the Greater London Area. Measurement locations are marked in squares.

The London site-specific air temperature prediction model (LSSAT) predicts on an hourly basis the air temperature at discreet locations within the city of London (based on input data from one meteorological station for the time the prediction is required and historic measured air temperatures within the city). It allows testing building performance compared to neighbourhood, city or regional weather (Figure 16) [99].

#### 3.3.7. EPA Mitigation Impact Screening Tool (MIST)

EPA Mitigation Impact Screening Tool (MIST) [101] is a software tool developed by the US Environmental Agency to provide an assessment of the impacts of several UHI mitigation strategies (mainly albedo and vegetation increase) on the reduction of the urban air temperatures, ozone and energy consumption for over 200 US cities [102]. The tool is currently unavailable, it was disabled by EPA due to the need to update the methodology and data inputs. Nevertheless, we have analysed how it functioned, as it attempted to provide a practical and customised assessment for the UHI reduction.

**Figure 17:**
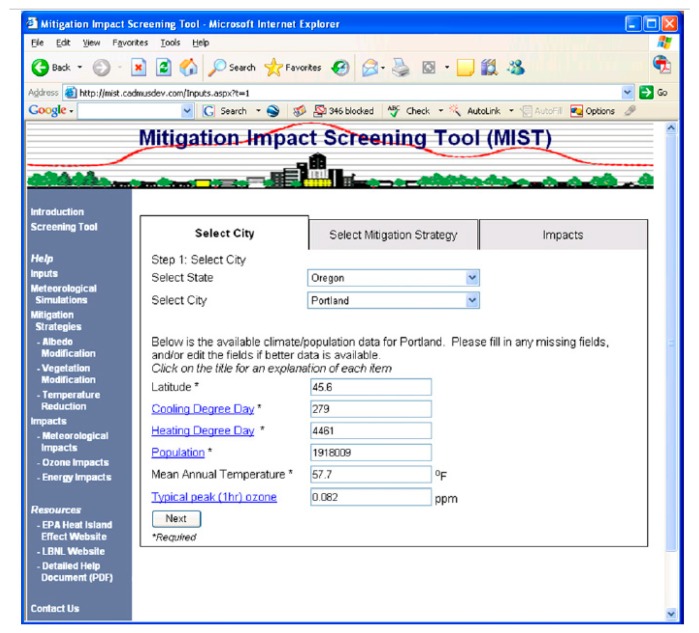
Screenshot of the input values that should be incorporated by the user, which refer to the selection of the city, and the corresponding city parameters (which can be adjusted manually).

As for most interactive tools, the first step is the selection of the city. The latitude, the cooling degree day (CDD), the heating degree day (HDD), the population, the mean annual temperature and the typical peak (one hour) ozone can be adjusted manually (Figure 17).

**Figure 18:**
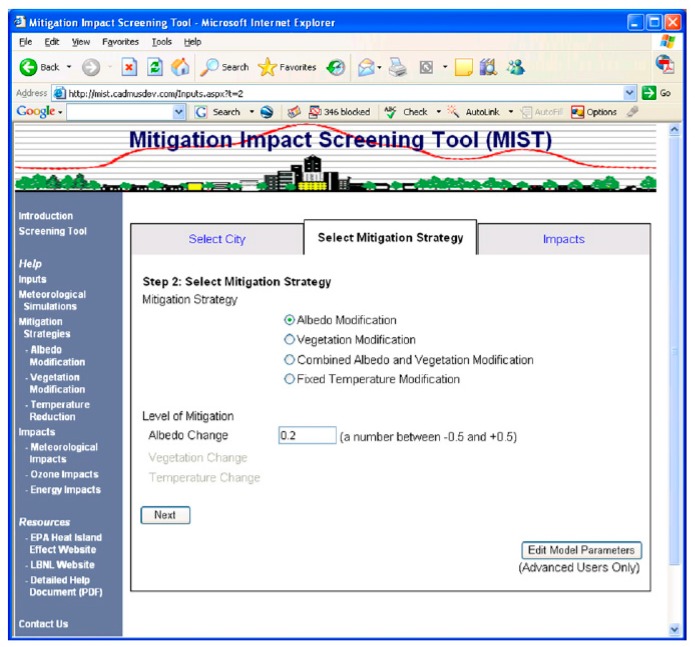
Screenshot of the mitigation strategy selected and its quantification. The mitigation strategy options are mainly albedo and vegetation modification or a combination of both.

The second step consists in the selection of the mitigation strategy and its quantification. The two options available consist in the modification of albedo and or the modification of the vegetation. The level of mitigation change is considered to be uniform across the selected city, it does not allow discriminating surfaces (roof tops from pavements), nor does it discriminate neighbourhood strategies within cities (Figure 18).

**Figure 19:**
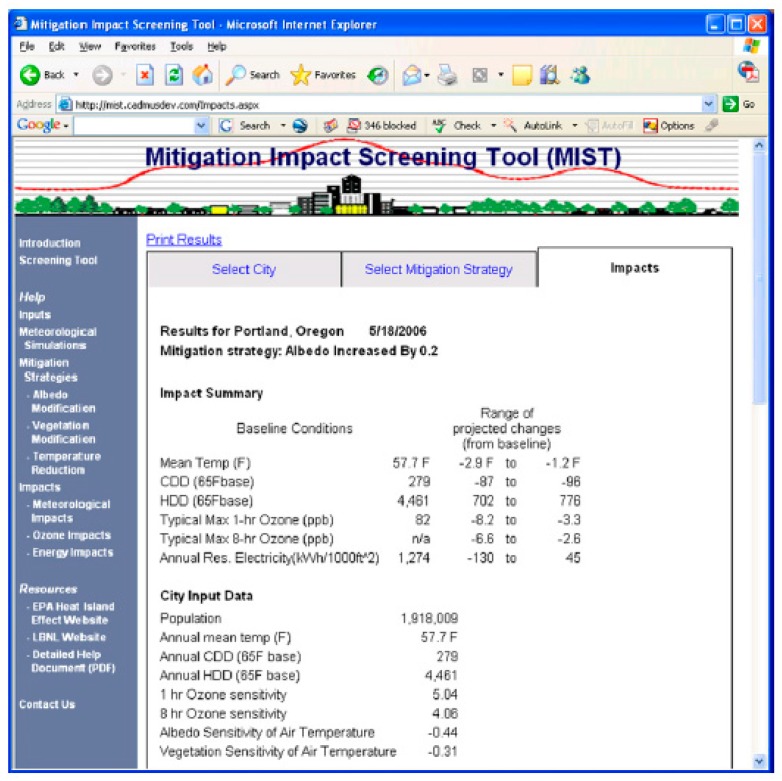
Screenshot of the impact estimation for the selected city and mitigation strategy.

The final step is the impacts estimation of the selected strategy in the selected city. The tool calculates the effect of the mitigation strategy on the reduction of the mean city temperature, the cooling degree days, the heating degree day, the typical 1 h and 8 h max ozone and on the energy consumption (Figure 19).

#### 3.3.8. The Urban Microclimate Tool

The Urban Microclimate tool is being developed at MIT, in order to control UHI to allow not only an increase of the thermal comfort but also a reduction of the energy use [103]. It will be integrated in Rhinoceros, thus allowing ensuring urban planners workflow continuity.

We have classified in four phases the type of assessment of the analysed tools (Table 2): phase 1 corresponds to the phase where the existing situation is analysed (current UHI mapped or mapping UHI related parameters), phase 2 corresponds to the analysis of the existing regulations or policies in force affecting UHI, phase 3 is the development of the concept design and phase 4 is the UHI assessment of the proposed conceptual design. Tools that provide mainly an assessment on phase 1 are actually databases that provide an assessment on UHI related parameters for a specific location. Tools that provide an assessment on phase 4 are simulation tools, which allow to test the efficiency of specific design proposals. Each of the tools requires different input parameters from the user (on the intervention to be tested) and provides different outputs and in different formats (urban heat island intensity, maximum surface temperature, applicable regulations).

These tools are valuable attempts to improve the understanding of the UHI for urban planners and decision makers, however the limitation of these tools is always geographical, as the accuracy of the assessment is directly connected to the quality of the input data, the resolution of the used maps and the complexity of the simulation models used. Another limitation of these digital tools, is that they often only provide an UHI assessment at one or maximum two of the design phases described above. A tool providing a comprehensive assessment from phase 1 till phase 4 is lacking.

Finally, each of the tools uses different input data and technologies (satellite imagery, meteorological stations measurements, meteorological model simulation algorithm…), and assess the user’s proposal based on: surface cover, volume and land use parameters.

Surprisingly none of the tools provide an estimation on the benefits of the implementation of the UHI mitigation measures on populations’ health, which would help decision makers the direct benefits of such actions on populations’ health.

## 4. Conclusions

A wide variety of cities—with different sizes, geographical characteristics, urban structures and in different climatological zones—are affected by the UHI phenomenon. The revision of related literature shows how broad the range of analysed parameters is—spatial contiguity, density, sprawl, storage heat flux, vegetation index, land surface temperature, albedo, sky view factor, coolspots, land use, imperviousness, social vulnerability and building vulnerability, cool wind paths,…—and how varied the suggested mitigation proposals are—increase of urban green, enhancement of peri-urban natural vegetation areas, consistent roof albedo modification in specific areas, maximising cooling properties of water bodies, raise social awareness, of influence the building and urban design.

In terms of health impacts of UHI, most of the health problems and fatal incidents take place during times of thermal extreme. In particular, persons with preexisting diseases, especially cardiovascular and respiratory diseases, are more often affected. In both sides of the scale, i.e., the very old, and the very young, are the most susceptible to such health problems. The effects of UHI on health are however difficult to quantify, because specific data about it is limited, and cases are poorly reported. Therefore, more attention should be paid to documentation in the occurrence as part of public health policies and measures.

Most of the UHI studies are site specific investigations, and the extrapolation of the results to other climate zones, geographies and urban areas might be challenging. In order to be able to provide a high level, first diagnosis on the phenomenon, several investigation groups have attempted to build UHI tool kits which provide a general UHI overview of the impact and potential solutions for specific regions, which can be taken into consideration by all parties involved in the process of construction and development of the urban environment (urban planners, politicians, economists, architects, teachers, citizens, …). However, these remain isolated cases, for specific locations and they do not quantify the effect of the measures on populations’ health.

As stated by Leal Filho et al. (2017) there is a need to better understand the phenomena of UHI and how it affects individual cities, and that there is a need to consider mitigation and adaptation strategies which take the particularities of each city into account so as to make them more resilient to UHI [104].

The increase of UHI and the implications on well-being and health presented in this article suggest that despite their different climatic, socioeconomic and public health status, the studied cities have been severely impacted over the last few decades, and might continue to be so in the near-future. The success of various adaptation measures will also be verified through the occurrence of UHI and this process needs closer monitoring. As far as future perspectives are concerned, it is important that:

(a) the current adaptation deficit be acknowledged and that cities actively try to address the problem they face right now
(b) a great emphasis to UHI be given in city development plans, so that the problem may be avoided or at least minimized in the future.

Ultimately, planners and designers need to recognise the scope and seriousness of UHI and ensure they are part of adaptation strategies.

## Figures and Tables

**Table 1:** Overview of a set of literature on UHI distributed by country.

Country	Reference	City	Air Temperature Difference	Summer	Winter	Period of Analysis	Parameters Analysed	Mitigation Measures Suggested
USA	National Center for Atmospheric Research, 2011 [55]		5.6					
	Hatchett et al. 2016 [56]	Reno, Nevada		X	X	1950–2014		
	Debbage & Shepherd, 2015 [57]	50 most populous cities in the US					Spatial contiguity, density and sprawl.	Spatial contiguity critical factor UHI. An increase of 10% in spatial contiguity might increase annual UHI by 0.3 and 0.4.
UK	Kershaw et al. 2010 [16]		0.1 to 1.9	X	X			
Belgium	Lauwaet et al. 2016 [32]	Brussels	3.15			2000–2009 and 2060–2069		
The Netherlands	Icaza et al. 2016 [19]	The Hague, Delft, Leiden, Gouda, Utrecht and Den Bosch		X			Storage heat flux, vegetation index, land surface temperature, albedo, sky view factor and coolspots.	Hotspots of 5 of the 6 cities were located in the seventeenth century City Center. Albedo interventions on those could reduce the effect by 1.5.
	Hove et al. 2011 [51]	The Hague, Delftand Leiden	4.8 and 5.6	X				
	Van der Hoven and Wandl 2015 [60]	Amsterdam	7	X			Land use, imperviousness, social vulnerability and building vulnerability.	
Germany	Office for Environmental Protection, section of urban climatology, 2008 [65]	Stuttgart					Cold production areas, air catchment areas and breeze systems.	Preservation and enhancement of existing green infrastructure surrounding the city.
Malaysia	Morris et al. 2015 [66]	Putrajaya	1.9 to 3.1				Vegetation surface	The overall effect of urbanised local climate zones is normalised by the total amount of area reserved for vegetation.
India	Borbora & Das 2014 [67]	Guwahati	>2				Green cover	The reduction of green cover associated with urbanisation, increases the UHI.
Japan	Fujibe 2011 [68]	Tokyo, Osaka and Nagoya		X			Surface heating over large surfaces, sea breeze penetration, temperature change evolution per decade, density.	

**Table 2:** Overview of different digital UHI tools available.

UHI Assessment TOOL	Geographical Cover	Scale of the Assessment	Type of Assessment	User Input Parameters	Tool Output
Decision Support System (DSS) UHI project	Bologna/Modena, Venice/Padua, Wien, Stuttgart, Lodz/Warsaw, Ljubljana, Budapest and Prague.	Supra-metropolitan	Phase 1: Mapping Urban Heat. Phase 2: Understanding regulations and policies related to UHI (greenery: street or roof, material reflectance…)	1/Location, 2/Scale (building or urban) 3/Typology of the intervention (building, facade, roofs, surface lots, urban structure and urban green) 4/Economic assessment 5/Skills.	1/climate change assessment (Change in annual mean temperature per decade, changes in annual near-surface temperature for 30 year periods and heat wave frequency), 2/a set of normative applicable to the selected area and skills, 3/a set of potential mitigation strategies.
CE Urban Heat Island Atlas UHI project	Central Europe region	Regional	Phase 1: Mapping Urban heat related parameters.	1/Location	1/Air temperature 2/Digital elevation models 3/Land surface temperature 4/Land cover regional scale (corine) 5/Urban land use.
STAR tools GRaBS project	North West region of England	Neighbourhood	Phase 4: Testing conceptual design	1/Location 2/Land cover proposal (% of buildings, major roads, other impervious surfaces, green and blue surfaces and bare soil or gravel surfaces) 3/Temperature scenario for 2050 (Baseline temperature, 2050’s 10% probability level, 50% probability level or 90% probability level).	1/Maximum surface temperature
London unified model (Londum)	City of London	City	Phase 1: Simluation map of the Urban Heat Island of the existing city. Phase 4: Simulation map of the Urban Heat Island of the projected city.	1/ Volume (Reflection, Shadowing, conduction of heat into the buildings, flux of heat into the atmosphere). Provided by the tool for the city of London.	1/Urban heat island intensity (air temperature at 1,5m height).
ADMS model	City of London	Neighbourhood	Phase 1: Simluation map of the Urban Heat Island of the existing city. Phase 4: Simulation map of the Urban Heat Island of the projected city.	1/Location 2/Surface cover (Albedo, evapotranspiration, thermal admittance).	2/Air temperature variations -due to land cover- at 2m height.
London site-specific air temperature prediction model (LSSAT)	City of London	Neighbourhood	Phase 1: Air temperature mapping at a particular time. Phase 4: Air temperature prediction based on intervention proposed.	1/Location	1/Hourly prediction of air temperature based on site specific transects (Global solar radiation, cloud cover, wind velocity and relative humidity).
EPA Mitigation Impact Screening Tool (MIST)	U.S.A. 230 cities	City	Phase 4: Testing the mitigation effect of the selected mitiation strategy.	1/Location 2/The latitude 3/the cooling degree day (CDD) 4/the heating degree day (HDD) 5/the population 6/the mean annual temperature 7/the typical peak (one hour) ozone 8/Mitigation strategy (albedo or vegetation modification).	Calculation of the effect of the mitigation strategy 1/Reduction of the mean city temperature 2/Cooling degree days 3/the heating degree day 4/the typical 1hr and 8hr max ozone 5/The energy consumption.

## References

[1] United Nations Department of Economic and Social Affairs (2014). World Urbanization Prospects: The 2014 Revision.

[2] United Nations (2016). Goal 11, Make Cities Inclusive, Safe, Resilient and Sustainable. http://www.un.org/sustainabledevelopment/cities/.

[3] Emmanuel R., Krüger E. (2012). Urban heat island and its impact on climate change resilience in a shrinking city: The case of Glasgow, UK. Build. Environ..

[4] Leal Filho W., Azeiteiro U., Alves F. (2016). Climate Change and Health: Improving Resilience and Reducing Risks.

[5] United States Centres of Disease Control and Prevention (CDC) (2012). Climate Change and Extreme Heat Events.

[6] Mills D., Schwartz J., Lee M., Sarofim M., Jones R., Lawson M., Duckworth M., Deck L. (2014). Climate change impacts on extreme temperature mortality in select metropolitan areas in the United States. Clim. Chang..

[7] Kleerekoper L., Van Esch M., Salcedo T.B. (2012). How to make a city climate-proof, addressing the urban heat island effect. Resour. Conserv. Recycl..

[8] Santamouris M. (2007). Heat Island research in Europe—The state of the art. J. Adv. Build. Energy Res..

[9] National Center for Atmospheric Research (UCAR) (2011). Urban Heat Islands. http://scied.ucar.edu/longcontent/urban-heat-islands.

[10] Roth M., Fernando H.J.S. (2013). Urban Heat Islands. Handbook of Environmental Fluid Dynamics.

[11] Chow W.T.L., Brennan D., Brazel A.J. (2012). Urban heat island research in Phoenix, Arizona. Bull. Am. Meteorol. Soc..

[12] National Geographic (2016). Urban Heat Island. NG Education Encyclopedia. http://education.nationalgeographic.org/encyclopedia/urban-heat-island/.

[13] Oke T.R. (1987). Boundary Layer Climates.

[14] IPCC (2001). Climate Change 2001, the Scientific Basis. Chapter 2.2 How Much is the World Warming?.

[15] Moreno-Garcia M. (1993). Carmen intensity and form of the urban heat island in barcelona. Int. J. Climatol..

[16] Kershaw T., Sanderson M., Coley D., Eames M. (2010). Estimation of the urban heat island for UK climate change projections. Build. Serv. Eng. Res. Technol..

[17] Baede A.P.M. (2007). Annex I Glossary. Climate Change 2007: The Physical Science Basis.

[18] Icaza L.E., van der Hoeven F., van den Dobbelsteen A. (2016). Surface thermal analysis of North Brabant cities and neighborhoods during heat waves. Tema J. Land Use Mobil. Environ..

[19] Icaza L.E., van den Dobbelsteen A., van der Hoeven F., Leal Filho W., Adamson K., Dunk R., Azeiteiro U.M., Illingworth S., Alves F. (2016). The Urban Heat Island Effect in Dutch City Centers: Identifying Relevant Indicators and First Explorations. Implementing Climate Change Adaptation in Cities and Communities.

[20] Icaza L.E., van den Dobbelsteen A., van der Hoeven F. (2016). Using satellite imagery analysis to redesign provincial parks for a better cooling effect on cities. The case study of South Holland. Research in Urbanism Series IV.

[21] Icaza L.E., van der Hoeven F. (2017). Regionalist principles to reduce the urban heat island effect. Sustainability.

[22] Péti M. Re-understanding Sustainability on Regional Level. Proceedings of the Role of Impact Assessment in Transitioning to the Green Economy 30th Annual Meeting of the International Association for Impact Assessment, International Conference Center.

[23] Cuevas S.C. (2011). Climate change, vulnerability, and risk linkages. Int. J. Clim. Chang. Strat. Manag..

[24] Mechler R., Hochrainer S., Aaheim A., Salen H., Wreford A. (2010). Modelling economic impacts and adaptation to extreme events: Insights from European case studies. Mitig. Adapt. Strat. Glob. Chang..

[25] Rosenzweig C., Solecki W.D., Hammer S.A., Mehrotra S. (2011). Climate Change and Cities (First Assessment Report of the Urban Climate Change Research Network).

[26] Hebbert M., Webb B. (2012). Towards a Liveable Urban Climate: Lessons from Stuttgart. Liveable Cities.

[27] Li D., Bou-Zeid E. (2013). Synergistic interactions between urban heat islands and heat waves: The impact in cities is larger than the sum of its parts. J. Appl. Meteorol. Clim..

[28] Li D., Sun T., Liu M., Yang L., Wang L., Gao Z. (2015). Contrasting responses of urban and rural surface energy budgets to heat waves explain synergies between urban heat islands and heat waves. Environ. Res. Lett..

[29] Founda D., Santamouris M. (2017). Synergies between urban heat island and heat waves in Athens (Greece), during an extremely hot summer 2012. Sci. Rep..

[30] US EPA (2015). Heat Island Impacts. https://www.epa.gov/heat-islands/heat-island-impacts.

[31] Keramitsoglou I., Sismanidis P., Analitis A., Butler T., Founda D., Giannakopoulos C., Giannatou E., Karali A., Katsouyanni K., Kendrovski V. (2017). Urban thermal risk reduction: Developing and implementing specifically explicit services for resilient cities. Sustain. Cities Soc..

[32] Lauwaet D., Hooyberghs H., Maiheu B., Lefebvre W., Driesen G., Van Looy S., De Ridder K. (2015). Detailed urban heat island projections for cities worldwide: Dynamical downscaling CMIP5 global climate models. Climate.

[33] Zhao L., Lee X., Smith R.B., Oleson K. (2014). Strong contributions of local background climate to urban heat islands. Nature.

[34] Kottek M., Grieser J., Beck C., Rudolf B., Rubel F. (2006). World map of Köppen-Geiger climate classification updated. Meteorol. Z..

[35] Steeneveld G.J., Koopmans S., Heusinkveld B.G., van Hove L.W.A., Holtslag A.A.M. (2011). Quantifying urban heat island effects and human comfort for cities of variable size and urban morphology in the Netherlands. J. Geophys. Res..

[36] Gonzalez-Aparicio I. (2012). Air Quality and Meteorological Modelling of Urban Areas in the Context of Climate Change.

[37] He J., Liu J., Zhuang D., Zhang W., Liu M.L. (2007). Assessing the effect of land use/land cover change on the change of urban heat island intensity. Theor. Appl. Climatol..

[38] Mohan M., Kikegawa Y., Gurjar B.R., Bhati S., Kolli N.R. (2013). Assessment of urban heat island effect for different land use—Land cover from micrometeorological measurements and remote sensing data for megacity Delhi. Theor. Appl. Climatol..

[39] Murphy D.J., Hall M.H., Hall C.A.S., Heisler G.M., Stehman S.V., Anselmi-Molina C. (2010). The relationship between land cover and the urban heat island in northeastern Puerto Rico. Int. J. Clim..

[40] Cai Y., Zhang H., Zheng P., Pan W. (2016). Quantifying the impact of land use/land cover changes on the urban heat island: A case study of the natural wetlands distribution area of Fuzhou City, China. Wetlands.

[41] NASA’s Goddard Space Flight Center. https://www.nasa.gov/vision/earth/environment/urban_effects.html.

[42] Mills G., Ching J., See L., Bechtel B., Foley M. An Introduction to the WUDAPT project. Proceedings of the 9th International Conference on Urban Climate.

[43] Bechtel B., Foley M., Mills G., Ching J., See L., Alexander P., O’Connor M., Albuquerque T., Andrade M., Brovelli M. CENSUS of Cities: LCZ Classification of Cities (Level 0)—Workflow and Initial Results from Various Cities. Proceedings of the ICUC9—9th International Conference on Urban Climate Jointly with 12th Symposium on the Urban Environment.

[44] Stewart I.D., Oke T.R. (2012). Local Climate Zones for Urban Temperature Studies. Bull. Am. Meteorol. Soc..

[45] (2014). STAR Joint Polar Satellite System. https://www.star.nesdis.noaa.gov/jpss/lst.php.

[46] Zhang P., Marc L., Imhoff M.L., Wolfe R.E., Bounoua L. (2014). Characterizing urban heat islands of global settlements using MODIS and nighttime lights products. Can. J. Remote Sens..

[47] NASA SEDAC (2013). Socioeconomic Data and Applications Center. http://sedac.ciesin.columbia.edu/data/set/sdei-global-uhi-2013.

[48] Oke T.R. (1973). City size and the urban heat island. Atmos. Environ..

[49] Park H.S. (1986). Features of the heat island in Seoul and its surrounding cities. Atmos. Environ..

[50] Fukuoka Y. (1983). Physical Climatological Discussion on Causal Factors of Urban Temperature. Memoirs Faculty Integrated Arts and Sciences.

[51] Hove L.W.A., Steeneveld G.J., Jacobs C.M.J., Heusinkveld B.G., Elbers J.A., Moors E.J., Holtslag A.A.M. (2011). Exploring the Urban Heat Island Intensity of Dutch Cities.

[52] Elsayed I.S.M. Effects of Population Density and Land Management on the Intensity of Urban Heat Islands: A Case Study on the City of Kuala Lumpur, Malaysia. http://dx.doi.org/10.5772/47943.

[53] Kotharkar R., Surawar M. (2016). Land use, land cover, and population density impact on the formation of canopy urban heat islands through traverse survey in the Nagpur urban area, India. J. Urban Plan. Dev..

[54] Stone B.J. (2007). Urban and rural temperature trends in proximity to large US cities: 1951–2000. Int. J. Climatol..

[55] US EPA (2016). Heat Island Effect. https://www.epa.gov/heat-islands.

[56] Hatchett B.J., Hatchett B.J., Koračin D., Mejía J.F., Boyle D.P. (2016). Assimilating urban heat island effects into climate projections. J. Arid Environ..

[57] Debbage N., Shepherd J.M. (2015). The urban heat island effect and city contiguity. Comput. Environ. Urban Syst..

[58] Wilby R.L. (2003). Past and projected trends in London’s urban heat island. Weather.

[59] Lauwaet D., de Ridder K., Saeed S., Brisson E., Chatterjee F., van Lipzig N.P.M., Maiheu B., Hooyberghs H. (2016). Urban climate assessing the current and future urban heat island of Brussels. Urban Clim..

[60] Van der Hoeven F., Wandl A. (2014). Amsterwarm: Mapping the landuse, health and energy-efficiency implications of the Amsterdam urban heat island. Build. Serv. Eng. Res. Technol..

[61] Founda D., Pierros F., Petrakis M., Zerefos C. (2015). Interdecadal variations and trends of the Urban Heat Island in Athens (Greece) and its response to heat waves. Atmos. Res..

[62] Kourtidis K., Georgoulias A.K., Rapsomanikis S., Amiridis V., Keramitsoglou I., Hooyberghs H., Maiheu B., Melas D. (2015). A study of the hourly variability of the urban heat island effect in the Greater Athens area during summer. Sci. Total Environ..

[63] Santamouris M., Paraponiaris K., Mihalakakou G. (2007). Estimating the ecological footprint of the heat island effect over Athens, Greece. Clim. Chang..

[64] Adapt (2015). Green Spaces and Corridors in Urban Areas. http://climate-adapt.eea.europa.eu/viewmeasure?ace_measure_id=4702.

[65] City of Stuttgart; Office for Environmental Protection; Section of Urban Climatology (2008). Climate Atlas of the Region of Stuttgart. http://www.stadtklimastuttgart.de/index.php?climate_climate_atlas_2008.

[66] Morris K.I., Salleh S.A., Chan A., Ooi M.C.G., Abakr Y.A., Oozeer M.Y., Duda M. (2015). Computational study of urban heat island of Putrajaya, Malaysia. Sustain. Cities Soc..

[67] Borbora J., Das A.K. (2014). Summertime urban heat island study for Guwahati City, India. Sustain. Cities Soc..

[68] Fujibe F. (2011). Urban warming in Japanese cities and its relation to climate change monitoring. Int. J. Climatol..

[69] Taylor A. (2016). Institutional inertia in a changing climate: Climate adaptation planning in Cape Town, South Africa. Int. J. Clim. Chang. Strateg. Manag..

[70] Rotem-Mindali O., Michael Y., Helman D., Lensky I.M. (2015). The role of local land-use on the urban heat island effect of Tel Aviv as assessed from satellite remote sensing. Appl. Geogr..

[71] Giguère M., Dubé N., Colas J. (2009). Urban Heat Island Mitigation Strategies.

[72] Commission de santé et de la sécurité au travail du Québec (CSST) (2004). Guide de Prévention des Coups de Chaleur.

[73] Raymond E.L., Bouchard A., Gagnon V. (2006). La Gestion du Risque de Chaleur Accablante ou Extrême dans L’agglomération de Montréal.

[74] Vienna University of Technology (2014). “Development and Application of Mitigation and Adaptation Strategies and Measures for Counteracting the Global Urban Heat Islands Phenomenon” (3CE292P3). http://www.central2013.eu/fileadmin/user_upload/Downloads/outputlib/UHI_Catalogue_of_Mitigation_and_Adaptation_strategies.pdf.

[75] Solecki W., Rosenzweig C., Parshall L., Pope G., Clark M., Cox J., Wiencke M. (2005). Mitigation of the heat island effect in urban New Jersey. Environ. Hazards.

[76] Hsieh C.M., Aramaki T., Hanaki K. (2007). Estimation of heat rejection based on the air conditioner use time and its mitigation from buildings in Taipei City. Build. Environ..

[77] Wen Y., Lian Z. (2009). Influence of air conditioners utilization on urban thermal environment. Appl. Therm. Eng..

[78] Yamamoto Y. (2006). Measures to Mitigate Urban Heat Islands. Quarterly Review.

[79] Cooling Singapore A Catalogue of Strategies to Mitigate Urban Heat Island and Improve Outdoor Thermal Comfort for Tropical Climate. https://www.coolingsingapore.sg/news-1/.

[80] Nuruzzaman M. (2015). Urban heat island: Causes, effects and mitigation measures—A review. Int. J. Environ. Monit. Anal..

[81] Li X.-X., Norford L.K. (2016). Evaluation of cool roof and vegetations in mitigating urban heat island in a tropical city, Singapore. Urban Clim..

[82] Susca T., Gaffin S.R., Dell’Osso G.R. (2011). Positive effects of vegetation: Urban heat island and green roofs. Environ. Pollut..

[83] Déoux S., Déoux P. (2004). Guide de l’Habitat Sain.

[84] Takebayashi H., Moriyama M. (2007). Surface heat budget on green roof and high reflection roof for mitigation of urban heat island. Build. Environ..

[85] Gago E.J., Roldan J., Pacheco-Torres R., Ordóñez J. (2013). The city and urban heat islands: A review of strategies to mitigate adverse effects. Renew. Sustain. Energy Rev..

[86] Santamouris M. (2013). Using cool pavements as a mitigation strategy to fight urban heat island—A review of the actual developments. Renew. Sustain. Energy Rev..

[87] Aleksandrowicz O., Vuckovic M., Kiesel K., Mahdavi A. (2017). Current trends in urb ban heat island mitigation research: Observations based on a comprehensive research repository. Urban Clim..

[88] European Environment Agency (EEA) (2012). Urban Adaptation to Climate Change in Europe (Challenges and Opportunities for Cities Together with Supportive National and European Policies). http://www.environment-agency.gov.uk/research/planning/127387.aspx.

[89] Barriopedro D., Fischer E.M., Luterbacher J., Trigo R.M., Garcia-Herrera R. (2011). The Hot summer of 2010, redrawing the temperature record map of Europe. Science.

[90] Burghardt R., Katzschner L., Kupski S., Chao R., Spit T. (2010). Urban Climatic Map of Arnhem City. Future Cities, Urban Networks to Face Climate Change. Interreg IV. www.future-cities.eu.

[91] City of Freiburg (2013). Land Use Plan 2020 of the City of Freiburg. http://planning.cityenergy.org.za/index.php/world-cities/europe/city-of-freiburg-germany.

[92] Hendel M., Gutierrez P., Royon L. (2016). Measuring the Effects of UHI Mitigation in the Field: Application to the Case of Pavement-Watering in Paris. Urban Clim..

[93] US EPA (2015). Adapting to Heat. https://www.epa.gov/heat-islands/adapting-heat#forecasting.

[94] Kiuila O., Wójtowicz K., Żylicz T., Kasek L. (2014). Economic and environmental effects of unilateral climate actions. Mitig. Adapt. Strat. Glob. Chang..

[95] Icaza L.E., van den Dobbelsteen A., van der Hoeven F. (2016). Integrating urban heat assessment in urban plans. Sustainability.

[96] Urban Heat Island Project (2016). Development and Application of Mitigation and Adaptation Strategies and Measures for Counteracting the Global Urban Heat Islands Phenomenon. http://eu-uhi.eu/project-focus/.

[97] STAR Tools (2016). Surface Temperature and Runoff Tools for Assessing the Potential of Green Infrastructure in Adapting Urban Areas to Climate Change. http://maps.merseyforest.org.uk/grabs/-.

[98] Davies M. LUCID. http://www.arcc-network.org.uk/wp-content/pdfs/ACN-LUCID1.pdf.

[99] Hamilton I., Davies M., Gauthier S. (2013). 2012, London’s Urban Heat Island: A Multi-Scaled Assessment Framework. Proc. Inst. Civ. Eng. Urban Des. Plan..

[100] Davies M. (2012). London Unified Model.

[101] (2016). Environmental Protection Agency. http://www.coolrooftoolkit.org/knowledgebase/epa-mitigation-impact-screening-tool-mist/.

[102] Sailor D.J., Dietsch N. (2007). The urban heat island Mitigation Impact Screening Tool (MIST). Environ. Model. Softw..

[103] MIT (2016). Urban Microclimate. UWG: Urban Design Software Development. http://urbanmicroclimate.scripts.mit.edu/umc.php.

[104] Leal Filho W., Icaza L.E., Neht A., Klavins M., Morgan E.A. (2017). Coping with the impacts of urban heat islands. A literature based study on understanding urban heat vulnerability and the need for resilience in cities in a global climate change context. J. Clean. Prod..

